# Prior information can alter how sounds are perceived and emotionally regulated

**DOI:** 10.1016/j.heliyon.2022.e09793

**Published:** 2022-06-24

**Authors:** Örn Kolbeinsson, Erkin Asutay, Johan Wallqvist, Hugo Hesser

**Affiliations:** a Linköping University, Sweden; b Örebro University, Sweden

**Keywords:** Verbal information, Auditory distraction, Sound perception, Emotion regulation, Suppression

## Abstract

In the current study, we provided participants with written information about emotional dimensions of a sound presented as a task-irrelevant sound in the context of a serial recall task. We were interested in whether this manipulation would influence sound perception and spontaneous use of emotion regulation strategies. Participants were informed that they would hear either an aversive and annoying sound, or a pleasant and calming sound. They subsequently performed three blocks of a serial recall task with the sound presented in the background and rated the sound after each block. Results showed that participants in the negative information group rated the sound as more negative, with effects diminishing over repeated trials. While not impacting emotion regulation strategy directly, the manipulation indirectly influenced the degree to which participants used mental suppression as a regulatory strategy via changing affective responses. In the negative information condition specifically, participants who experienced the sound as more negative were more inclined to use mental suppression to deal with the sound, whereas no such relationship was observed in the positive information condition. The study adds to our understanding of how sounds come to acquire emotional meaning and how individuals spontaneously cope with emotional, task-irrelevant sounds.

## 1 Prior information can alter how sounds are perceived and emotionally regulated

Emotion can have a powerful impact on perceptual and attentional processes (e.g., Vuilleumier, 2005; Zadra and Clore, 2011). Studies on emotional perception and attention have mainly been conducted with visual stimuli, but recent findings suggest that similar effects can be shown in the auditory domain. For example, emotionally negative and arousing sounds can affect attention by facilitating change detection in a change deafness paradigm (Asutay and Västfjäll, 2014), and negative mood impacts perceptions of loudness (Siegel and Stefanucci, 2011) and annoyance (Västfjäll, 2002) in response to sound.

While certain sound characteristics such as high intensity or sharpness will inherently elicit an emotional response (Västfjäll, 2012), many sounds evoke emotions by virtue of their significance in a person's current or historical context (Juslin and Västfjäll, 2008). The importance of the learned meaning of sounds has recently received increased interest and has been shown to be integral to understanding how sounds gain emotional significance (Asutay et al., 2012). In an experimental setting, this is most commonly studied using classical conditioning procedures where a neutral sound is repeatedly paired with an aversive or appetitive stimulus (Asutay and Västfjäll, 2017). Research using these procedures have reliably shown effects on sound perception: Conditioning fear to a sound has, for example, been shown to affect pitch discrimination for that sound (Resnik et al., 2011) and can also cause a sound to be perceived as louder and more threatening (Asutay and Västfjäll, 2012). In addition to classical conditioning procedures, research suggests that emotional and evaluative responses can be established by providing verbal instructions (Mertens et al., 2018). Indeed, simply informing individuals about the emotional significance of upcoming events or stimuli can be sufficient to change emotional reactions towards said stimuli. While changes in emotional and evaluative responses following such procedures have been documented with a range of stimuli, most visual (see Mertens et al., 2018), we are aware of no study examining whether providing emotional information about a sound can alter associated emotional, perceptual and behavioral responses.

In addition to influencing perception, emotionally significant sounds are powerful attractors of attention (Bröckelmann et al., 2011). This has natural benefits when the sound is relevant to the task at hand (e.g., Asutay and Västfjäll, 2014; Asutay and Västfjäll, 2017), or when the sound carries information about a potential reward (Asutay and Västfjäll, 2016), but can be detrimental when an emotional sound is irrelevant to task performance (Max et al., 2015). When an emotional stimulus threatens to disrupt performance on an ongoing task, it will readily evoke a regulatory response (Gross, 1998a). This implies that any investigation into the determinants of emotional distraction must also account for participants’ attempts to regulate their emotions. Emotion regulation has been conceptualized as the process by which people modulate their emotional responses to meet the demands of the environment (Gross, 1998b). In the emotion regulation literature, humans are often described as having a number of different strategies they may use for regulating their emotions (e.g., suppression, acceptance, reappraisal, etc.; Gross 1998a; 1998b). The choice of emotion regulation strategy has been shown to depend, among other things, on affective arousal and motivational factors (Dixon-Gordon et al., 2015; Sheppes et al., 2011). Additionally, while the recurrent use of specific emotion regulation strategies appears to be more or less maladaptive, there is evidence to suggest that the effectiveness of strategies is also related to contextual demands (Aldao et al., 2010; Webb et al., 2012).

Although emotion regulation has been extensively studied (e.g., Aldao et al., 2010), little is known about the use and impact of emotion regulation strategies in response to non-musical auditory stimuli. A noteworthy exception is a study by Hesser et al. (2013) showing that mental suppression had delayed costs in the form of reduced perseverance on a subsequent mentally challenging task. Specifically, participants who were instructed to suppress an aversive, high-pitched tone in an initial phase were less persistent than their counterparts in a control condition when performing a persistence task in the presence of the same sound. The finding is consistent with evidence indicating that suppression, as a mental or emotional control strategy, has detrimental effects on cognitive performance measures (for a review, see Wang et al., 2020). The authors further showed that a counterinduction in the form of a mindfulness exercise mitigated the counterproductive effects of suppression. Mindfulness is strongly related to another commonly studied emotion regulation strategy, namely *acceptance*. Acceptance, in this context, is the active action of allowing internal sensations and reactions without judgment and without attempts at reduction or control (Hayes et al., 1999). The results are consistent with findings from other domains (Sheppes et al., 2011, 2014), suggesting that while disengaging strategies, such as distraction or mental suppression, may be effective in the short term, they may be inferior in the long term.

As in Hesser et al. (2013), participants in emotion regulation studies are often instructed to use a specific regulatory strategy in order to determine its effectiveness in a particular context. Other studies have focused on the choice of strategy by giving participants a choice between several strategies in response to an emotional stimulus or situation (e.g., Sheppes et al., 2011). Feldman and Freitas (2021), for instance, studied the effects of emotional intensity on choice of emotion regulation strategy. The authors found that when choosing between *distraction* and *reappraisal*, participants preferred distraction when intensity was high, but reappraisal when intensity was low. Results were similar for visual and auditory stimuli, suggesting that choice of emotion regulation strategy is similar across stimulus modalities.

While studies on predetermined strategies have garnered important insights, some researchers have argued for the importance of studying spontaneous use of emotion regulation strategies (e.g., Aldao et al., 2010). As with studies on emotion regulation choice, studies on spontaneous emotion regulation suggest that emotional valence and intensity are important determinants of the emotion regulation strategies participants prefer to use (Dixon-Gordon et al., 2015; Szasz et al., 2018). For example, Dixon-Gordon et al. (2015) found that while certain strategies, such as acceptance and reappraisal, were among the most common strategies in general, strategy choice was also determined by type of emotion and emotional intensity. Furthermore, recent studies have suggested that it may be misleading to construe emotion regulation as involving one strategy at a time (Opitz et al., 2015; Szasz et al., 2018). Indeed, Opitz et al. (2015) showed that even when instructed to use one particular strategy, participants are likely to use additional supplementary strategies to optimize their regulatory response. Thus, it is important to investigate the simultaneous use of multiple strategies. The current study is, to our knowledge, the first to investigate spontaneous, uninstructed emotion regulation in response to non-musical sound stimuli.

The primary aims of the current study were twofold: First, to investigate whether verbal information (positive or negative) about a sound's emotional properties would alter the sound's emotional significance as evidenced by subjective ratings of the sound on affective dimensions and perceived loudness. Second, to study the emotion regulation strategies that participants spontaneously employed and investigate their relation to subjective ratings of the sound on affective dimensions. To frame the sound as potentially disruptive, and thus increase the relevance of using regulatory strategies, we implemented the verbal manipulation in the context of a serial recall task commonly used to study auditory distraction (e.g., Jones and Macken, 1993; Röer et al., 2017). Specifically, we took inspiration from a study by Röer et al. (2017) where the authors used a verbal manipulation about a sound's ability to distract attention. In our study, the chosen sound was an emotionally neutral noise (i.e., band pass filtered pink noise), which, in and of itself, was not expected to disrupt recall performance. The purpose of the task was, therefore, not primarily to elicit distraction but rather to create a context so that participants would be inclined to employ regulatory strategies in response to the sound. The particular sound was chosen because it has previously been shown that verbal information can alter evaluative responses toward similar noises (Bergman et al., 2008). Most importantly for the current study, the sound is not inherently pleasant nor unpleasant, and both negative and positive information about the sound should appear believable to participants. We predicted that presenting negative information about the sound would result in participants rating the sound as having more negative valence, being more arousing and annoying. As negative emotion has previously been related to increased loudness perception (Asutay and Västfjäll, 2012), we expect participants in the negative information condition to rate the sound as louder. In line with previous research on emotion regulation (Barrett et al., 2001; Dixon-Gordon et al., 2015), we predicted that the negative information manipulation would result in the participants being more inclined to employ emotion regulation strategies generally. In particular, we hypothesized that they would report greater use of strategies associated with disengaging from the sound (i.e., suppression, distraction), as previous studies suggest that these strategies are more likely to be endorsed when a stimulus is more negative and more intense.

## 2 Method

### 2.1 Participants

A total of 81 participants were recruited on a university campus. One participant was subsequently excluded due to an inability to comply with the instructions. In the final sample, 37 participants reported that they identified as female, 41 as male, and 2 as non-binary. Participants were aged between 18 and 58 years (*M* = 25.66). Recruitment was carried out by distributing leaflets on campus and by sending information to university students and staff who had expressed interest in participating in psychological research. Participants were randomly assigned to either a *negative information* (19 female, 20 male, 1 non-binary; mean age = 24.85) or *positive information* condition (18 female, 21 male, 1 non-binary; mean age = 26.48). Criteria for participating were: adequate proficiency in Swedish, normal or corrected to normal vision, normal hearing, and no perceived issues with regards to tinnitus or hyperacusis. All participants received a gift worth a maximum of SEK 100 as compensation for their time. The experiment was approved by the Swedish Ethical Review Authority (EPN DNR, 2014/162-31, 2018/90-32, 2018-419-31, 2019-01038, 2021-05673-02), and was conducted in accordance with the ethical principles outlined in the Declaration of Helsinki for human studies. Data were stored anonymously. All participants provided informed consent by signing a written consent form.

### 2.2 Materials

#### 2.2.1 Serial recall task

On each trial of the serial recall task, participants were presented with eight to-be-remembered digits sampled without replacement from the set *1–9*. Digits were presented in the middle of the screen in white font against a black background, subtending a visual angle of 3°. One digit was presented per second, the digit being visible for 800 m with a subsequent inter-stimulus interval of 200 m. Immediately after the final digit had been presented, participants were prompted to input the digits in the same order as they had been presented. Each trial lasted approximately 20 s. During the testing phase, the sound was presented on 4 out of the 8 trials in each block, while the remaining trials were completed in silence. The task-irrelevant noise was presented during the stimulus presentation phase of the serial recall task. Sound onset was simultaneous to the onset of the first digit, and sound offset was simultaneous to the offset of the final digit. The sound was thus presented for a duration of 8 s on each serial recall trial. When choosing the sound for the current study, it was deemed necessary that the sound could plausibly be perceived as both negative and positive. Västfjäll (2012) has previously shown that synthetic noise with low sharpness (i.e., low high-frequency content) presented at an acceptable loudness will be perceived as approximately neutral on the emotional dimensions of arousal and valence. Pink noise was chosen as a basis for the sound, as it has been shown that the perceived valence of pink noise can be altered by pairing with positively or negatively valent cues (Bergman et al., 2008). The sound was thus constructed by applying a low-pass filter with a cut-off frequency of 220.00Hz, and a high-pass filter with a cut-off frequency of 293.66Hz to a broadband pink noise. Both filters had a roll-off of 6dB per octave. The sound was presented binaurally through a set of Beyerdynamic DT 770 headphones at a level of 68dB(A). The sound level was calibrated using a Bruel & Kjaer 2250 sound level meter and a Larson Davis 2541 microphone placed inside an artificial ear.

#### 2.2.2 Self-report measures

Participants were asked to rate the sound on four dimensions: *valence* (negative vs. positive), *arousal* (calming vs. agitating), *annoyance* (not at all vs. extremely annoying), and *loudness* (barely audible vs. extremely loud). Each time the sound was rated, participants were prompted to listen closely to the sound for five seconds, whereafter questions pertaining to the four dimensions were presented, one at a time, in random order. They rated the sound on a visual analog scale (VAS), using the computer mouse to position a marker on a horizontal line between two anchors (0, 100).

Finally, participants were asked to fill in a modified version of the State Emotion Regulation Inventory (SERI), a self-report measure developed to assess momentary usage of emotion regulation strategies (Katz et al., 2017). The original SERI consists of 16 items divided into 4 subscales measuring participants’ tendencies to use emotion regulation strategies related to *distraction*, *brooding*, *reappraisal* or *acceptance*. The questionnaire was modified to assess strategies used to regulate emotion elicited by the task-irrelevant sound. Since items pertaining to brooding did not appear relevant in the current context, these were replaced with four questions about mentally *suppressing* the sound (see the revised form in Supplemental Materials). The internal consistencies of the subscales for our sample were: suppression α = 0.82, acceptance α = 0.84, reappraisal α = 0.84 and distraction α = 0.62. Thus, according to conventional standards, internal consistency was adequate for suppression, acceptance, and reappraisal, and below adequate for distraction.

### 2.3 Procedure

Participants completed the experiment individually in a sound-attenuated room. Upon arrival, participants were briefed about the experiment and presented with a written consent form. Participants initially completed 5 trials of serial recall in silence to familiarize them with the task. Participants were then informed that they would perform the serial recall task again, this time with a sound present in the background. The experiment was identical for both groups, apart from the wording of the information regarding the auditory task-irrelevant sound. This difference constituted the manipulation in the experiment and concerned the emotional effect that the noise would have on participants during the serial recall task. Participants in the negative information condition received the following instructions:

You will now perform the same task again. This time you will hear a sound through the headphones on some trials while you are performing the task. The sound is not relevant to the task, and you should ignore it. The sound you are going to hear has been shown to be very irritating and is perceived by many as unpleasant. Previous studies also suggest that concentrating is more difficult while this type of sound is present in the background.

Participants in the positive information condition received identical instructions except for the two last sentences, which read:

The sound you are going to hear has been shown to have a calming effect and is perceived by many as pleasant. Previous studies also suggest that concentrating is easier while this type of sound is present in the background.

After receiving the instructions, participants performed three blocks of serial recall, each block consisting of eight trials. The task-irrelevant sound was presented on half of the trials in each block, while the remaining half of the trials were performed in quiet. After each block, participants rated the sound on dimensions of valence, arousal, annoyance, and loudness using a VAS. Finally, participants were asked to fill out a pen-and-paper version of the modified SERI-scales. The total duration of the experiment was between 20 to 25 min.

### 2.4 Data analysis

All statistical analyses were performed using *R statistical software* (R Core Team, 2021) and ANOVAs were conducted using the *afex* package (Singmann et al., 2021). The dependent variables were ratings of the sound, serial recall performance, and use of emotion regulation strategies. To determine the effect of the manipulation on ratings of the sound, we conducted four 2 (negative vs. positive information) × 3 (first vs. second vs. third phase) mixed-design ANOVAs. To test whether the manipulation had an effect on serial recall performance, a 2 (negative vs. positive information) × 2 (sound vs. no sound) × 3 (first vs. second vs. third phase) mixed design ANOVA was conducted. For the SERI-ratings, we conducted a 2 (negative vs. positive information) × 4 (suppression vs. acceptance vs. reappraisal vs. distraction) mixed-design ANOVA. In case of violated sphericity assumption, a Greenhouse-Geisser correction was applied. Furthermore, correlations between sound ratings and emotion regulation strategies were conducted to investigate the relationship between sound perceptions and emotion regulation strategy.

Finally, to formally evaluate whether the verbal manipulation would impact the use of emotion regulation strategies through changes in affective responses, we tested for mediation using the *mediation* package (Tingley et al., 2014), which provides causally defined direct and indirect effects within the potential outcomes framework (Imai et al., 2010). The mediation model thus consisted of information condition (positive/negative information) as the exposure variable, subjective ratings of affective response as the mediator, and the use of a particular emotion regulation strategy as the outcome variable. Linear regression models for both the mediator and outcome were fitted separately to estimate the effect of the exposure variable on the mediator (*a*-path), of the effect of the mediator on the outcome variable (*b*-path), the total effect of the exposure variable on the outcome variable (*c*-path), and the direct effect of the exposure variable on the outcome variable (*c’*-path). The models were subsequently combined to estimate the indirect effect of the exposure variable on the outcome via the mediator, defined as the product of the *a-*path and *b-*path (*ab*-path). Based on recent recommendations for evaluating mediation (Valeri and VanderWeele, 2013), we allowed the effect of the mediator on the outcome to vary as a function of experimental condition and fitted a moderated mediation model. This was done by including the interaction term between the exposure variable and mediator in the outcome model, and calculating conditional indirect effects using the *mediation* package. This procedure returns one indirect (and direct) effect per condition derived from parameter estimates, including the interaction term. For the negative information condition (coded as 1), the indirect effect was computed based on the sum of the *ab*-product and the interaction effect, whereas for the positive information condition the indirect effect is simply the *ab*-product (see for further technical details, MacKinnon et al., 2020; Tingley et al., 2014). Significance testing for mediation was done using bias-corrected and accelerated 95% confidence intervals from 3000 bootstrapped samples drawn with replacement.

## 3 Results

### 3.1 Effects on subjective sound ratings and serial recall

Means and standard deviations for subjective sound ratings across conditions are presented in Table 1. For ratings of valence, there was a statistically significant main effect of information condition, *F*(1, 78) = 5.26, *p* = .024, ${\hat{\eta }}_{p}^{2}$ = .063, and a two-way interaction between time and information condition, *F*(1.96, 151.15) = 5.30 *p* = .009, ${\hat{\eta }}_{p}^{2}$ = .064. These results reflect that participants in the negative information condition generally rated the sound as more negative than participants in the positive information condition and that this effect was stronger during the initial phases of the experiment. No main or interaction effects involving information condition reached statistical significance for ratings of arousal, annoyance, or loudness (all F < 1.19, all p > .30).

**Table 1:** Descriptive statistics for subjective sound ratings.

	Block	Pooled		Negative information		Positive information	
		M	SD	M	SD	M	SD
Valence							
	First	51.03	17.50	44.87	15.91	57.19	17.02
	Second	50.45	15.85	47.71	15.99	53.19	15.43
	Third	48.70	14.40	47.15	15.36	50.24	13.39
Arousal							
	First	34.33	20.53	36.67	21.40	31.99	19.61
	Second	39.60	20.86	39.71	21.42	39.50	20.55
	Third	41.17	20.60	41.64	20.95	40.70	20.49
Annoyance							
	First	40.02	25.54	42.89	26.65	37.15	24.37
	Second	42.78	22.60	44.74	22.88	40.82	22.43
	Third	44.45	21.71	45.51	22.40	43.39	21.23
Loudness							
	First	54.07	15.22	52.52	14.94	55.62	15.52
	Second	56.90	12.93	55.60	14.46	58.20	11.23
	Third	56.40	12.19	55.38	12.48	57.43	11.97

*Note.* Means and standard deviations pooled and as a function of experimental conditions for the subjective sound ratings.

Means and standard deviations related to serial recall performance are presented in Table 2. The ANOVA for serial recall performance indicated no main effects of either information condition, *F*(1, 78) = 0.28, *p* = .599, ${\hat{\eta }}_{p}^{2}$ = .004 or sound condition, *F*(1, 78) = 1.49, *p* = .226, ${\hat{\eta }}_{p}^{2}$ = .019. A significant main effect was found for time, *F*(1.96, 153.06) = 8.38, *p* < .001, ${\hat{\eta }}_{p}^{2}$ = .097. Contrasts showed that there was a significant improvement in performance between the first block and the second block, *t*(78) = -2.96, *p* = .008, but no improvement between the second and the third block, *t*(78) = -0.99, *p* = .586. None of the interaction effects approached significance.

**Table 2:** Descriptive statistics for serial recall performance by condition.

Block	Pooled		Negative information		Positive information	
	M	SD	M	SD	M	SD
First	0.55	0.27	0.56	0.26	0.53	0.29
Second	0.59	0.27	0.59	0.28	0.59	0.26
Third	0.60	0.27	0.62	0.27	0.59	0.27

*Note.* Means and standard deviations for proportion of correctly recalled items as a function of experimental condition.

### 3.2 Effects on spontaneous use of emotion regulation strategies

Descriptive statistics for the SERI across conditions are presented in Table 3. In summary, all four regulation strategies from the revised SERI were employed to some degree by members of both information conditions. The strategy most highly rated was acceptance (*M* = 22.38, *SD* = 5.22), followed by distraction (*M* = 19.21, *SD* = 5.43), suppression (*M* = 17.03, *SD* = 7.45) and reappraisal (*M* = 15.00, *SD* = 6.63). The mixed-design ANOVA for SERI-ratings revealed a significant main effect of strategy, *F*(2.58, 201.20) = 23.32, *p* < .001, ${\hat{\eta }}_{p}^{2}$ = .230, but no effect of condition *F*(1, 78) = 1.44, *p* = .234, ${\hat{\eta }}_{p}^{2}$ = .018, and no interaction effect between strategy and information condition, *F*(2.58, 201.20) = 0.65, *p* = .559, ${\hat{\eta }}_{p}^{2}$ = .008. Pairwise comparisons revealed that acceptance was significantly more common than distraction, *t*(79) = 3.39, *p* = .001, *d* = 0.38, distraction was significantly more common than suppression, *t*(79) = 3.22, *p* = .002, *d* = 0.36, and finally, suppression was significantly more common than reappraisal, *t*(79) = 2.24, *p* = .028, *d* = 0.25.

**Table 3:** Descriptive statistics for the emotion regulation questionnaire.

Variable	Pooled		Negative information		Positive information	
	M	SD	M	SD	M	SD
SERI Suppression	17.34	6.67	16.95	7.11	17.73	6.27
SERI Acceptance	22.34	5.10	23.10	4.57	21.58	5.54
SERI Reappraisal	14.90	6.57	15.60	7.12	14.20	5.97
SERI Distraction	19.68	4.99	20.18	4.97	19.18	5.02

*Note.* Means and standard deviations for the SERI, pooled and across experimental conditions.

### 3.3 Associations between emotion regulation strategies and affect

To explore associations between subjective ratings of affective response and emotion regulation strategies, a series of correlations were run. Correlations were analyzed separately by condition and are presented in Table 4. The affective ratings of valence, arousal, and annoyance were highly correlated in both information conditions. Loudness was significantly related to all affective dimensions in the negative information condition but only related to arousal in the positive information condition. Regarding the use of emotion regulation strategies, increased annoyance was significantly related to lesser use of acceptance strategies and more use of mental suppression in the positive information condition. Additionally, increased arousal was also related to increased use of mental suppression. In the negative information condition, increased annoyance was related to more use of distraction, and more negative valence was related to more use of mental suppression.

**Table 4:** Correlations between emotion regulation strategies and subjective sound ratings as a function of experimental conditions.

Variable		1	2	3	4	5	6	7	8
		**Negative information**							
1. Acceptance	**Positive information**		-.05	-.15	-.13	-.27	-.38	.15	-.15
2. Distraction		.07		.44∗∗	.12	.33∗	.13	-.30	-.06
3. Suppression		-.24	.39∗		-.11	.27	.05	-.40∗	.02
4. Reappraisal		-.27	-.02	-.03		.01	.01	.10	-.16
5. Annoyance		-.48∗∗	.08	.37∗	.01		.85∗∗	-.80∗∗	.51∗∗∗
6. Arousal		-.23	.21	.34∗	-.08	.51∗∗		-.57∗∗	.55∗∗
7. Valence		.05	-.03	-.12	.14	-.49∗∗	-.50∗∗		-.53∗∗
8. Loudness		-.06	-.07	.11	.24	.30	.37∗	-.08	

*Note.* Coefficients below the diagonal relate to the positive information condition and coefficients above the diagonal relate to the negative information condition.

∗ indicates *p* < .05.

∗∗ indicates *p* < .01.

∗∗∗ indicates *p* < .001.

**Figure 1:**
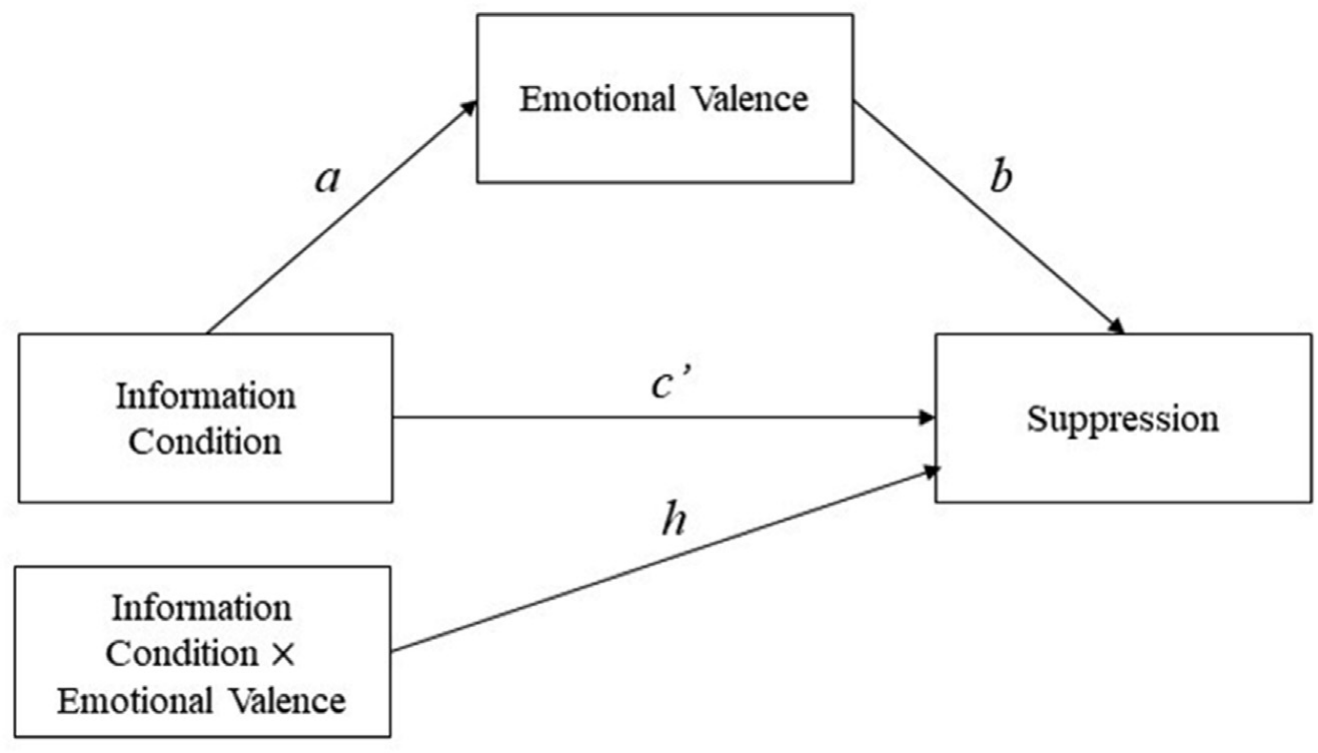
Statistical schematic of the mediation model. Note. The statistical model showing the effect of information condition on use of suppression as mediated by emotional valence. Furthermore, the effect of emotional valence on use of suppression was allowed to vary as a function of information condition.

Given the effect of information condition on ratings of valence, and the relationship between valence and use of mental suppression, we formally tested the mediated effect of information condition on use of mental suppression via subjective ratings of valence. As there were differences in associations as a function of condition (b-paths), the effect of valence on suppression was allowed to vary as a function of information condition (see Figure 1). In line with earlier findings, there was a significant indirect effect of negative valence on the use of mental suppression in the negative information condition, indirect effect = 1.43, 95% CI [0.12, 3.31] but not in the positive condition, indirect effect = 0.40, 95% CI [−0.66, 1.76].

## 4 Discussion

In the present experiment, we presented participants with written information about a sound's emotional aspects in an attempt to influence participants' perceptions of that sound. Results show that ratings of the sound's valence were influenced in the expected direction. Specifically, participants who were informed that they would hear a pleasant and calming sound rated the sound as more pleasant, and participants informed that they would hear an unpleasant and arousing sound rated the sound as more unpleasant. No statistically significant effects were found on ratings of either arousal, loudness, or annoyance. The effects were strongest immediately after the manipulation and diminished over subsequent trials. Furthermore, we found that while the manipulation did not directly influence participants' use of emotion regulation strategies, there was an indirect effect of information condition on the use of mental suppression, mediated by the effect of the manipulation on ratings of emotional valence. Specifically, in the negative information condition, increased negative valence was associated with an increased inclination to use mental suppression. These findings suggest that brief, written information about sounds can influence how such stimuli are perceived and evaluated in terms of emotional significance, and that this, in turn, will determine the strategies used to regulate emotion in the presence of distracting sound. The study adds to and expands on the growing literature on how prior information can alter the emotional significance of task-irrelevant sound in the context of auditory distraction (e.g., Röer et al., 2017), and on how individuals spontaneously use regulatory strategies in response to emotional stimuli (e.g., Szasz et al., 2018).

The role of verbal instructions in psychological experiments is likely understated. Studies on using verbal instructions to condition fear have shown how powerful verbal information can be in eliciting subjective, physiological, and behavioral fear responses (e.g., Mertens et al., 2018). A review by Mertens et al. (2018) shows that the moderating effects of verbal instructions have been obtained for a wide variety of conditioned responses, a wide variety of conditioning procedures and have been found in different labs and by different researchers. Together, these findings speak to the robustness of the effects of verbal instructions on fear conditioning via CS-US pairings. Typically, these verbal instructions describe the contingency between the neutral stimulus and an emotionally salient stimulus. In contrast, the manipulation used in the current study informed participants that the sound itself *was* an emotional stimulus without any further reference to other stimuli. Our manipulation also aimed to change perceived pleasantness, not induce fear. As such, our procedure is more akin to methods used to alter evaluative responses (e.g., Hofmann et al., 2010; Hughes et al., 2016). Indeed, verbal information has been shown to change likes and dislikes, although such procedures have not yet been used to change reactions toward auditory stimuli. Although the underlying mechanisms by which verbal information produces change in evaluative responses are debated – or even whether such procedures constitute evaluative conditioning (Hughes et al., 2016) – verbal instructions or other types of verbal information may have an impact on evaluative responses by derived symbolic meaning (De Houwer and Hughes, 2016). In fact, evaluative conditioning has more recently been proposed to be a symbolic phenomenon, suggesting that verbal processes may be important for a range of observed effects in evaluative conditioning research more generally (De Houwer and Hughes, 2016; Hughes et al., 2016).

In contrast to the effects of the manipulation on ratings of valence, no effects were found for ratings of arousal, loudness, or annoyance. This is most notable in regard to loudness, as previous studies have shown a link between negative affect and perceptions of loudness in both a mood induction paradigm (Siegel and Stefanucci, 2011) and a conditioning paradigm (Asutay and Västfjäll, 2012). This may suggest different pathways by which emotions can come to influence perceptions of loudness. It is possible, for instance, that the relationship between affect and sound intensity is mediated by increased physiological arousal. In previous studies by Asutay and Västfjäll (2017), as well as Parker et al. (2012), participants were made to experience fear, which is generally associated with a measurable physiological response. The current manipulation may not have resulted in a substantial physiological response, which could be one explanation for why we did not see any apparent effects on loudness. More research is warranted to examine, in greater detail, the link between emotional responding, including behavioral and physiological responses, and the perception of loudness.

Regarding emotion regulation strategies, the manipulation did not directly affect the degree to which participants attempted to regulate their emotions or the strategies they used. Choice of regulation strategy was, however, related to participants’ emotional response to the sound, and these relationships differed between participants in the two conditions. Furthermore, there was a statistically significant indirect effect of information condition on mental suppression via change in affective responses. Specifically, the manipulation influenced subjective ratings of emotional valence, and negative emotional valence was, in turn, related to greater use of mental suppression – but only in the negative information condition. Thus, it is noteworthy that the manipulation, by changing the verbal context, also seemed to change the mediator-outcome association (commonly assumed to be homogenous across conditions in classic mediation analysis; MacKinnon et al., 2020). This finding suggests that the specific verbal context (negative vs. positive) in which ratings of affective responses are made can potentially alter the meaning of such ratings, which in turn can influence the inclination to employ certain emotion regulation strategies. It is important to note that while we specified emotional valence as the mediator in our model, our data do not allow us to draw conclusions about the directionality of the effect between emotional valence and the use of mental suppression. The current manipulation was aimed specifically at altering perceptions of emotional valence, and previous studies suggest that choice of regulatory strategy is partially determined by emotional stimulus characteristics (e.g., Feldman and Freitas, 2021; Sheppes et al., 2014), lending some support for our proposed model. It is, however, probable that the relationship is bidirectional and that the use of a particular regulatory strategy will also impact stimulus evaluation. While the current study provides initial results on one way to model this relationship, future studies should work to elucidate this possibly dynamic association between emotion and regulatory strategy in the context of auditory stimuli. These limitations notwithstanding, our results give further credence to the notion that the adaptiveness of emotion regulation is contextually determined (e.g., Gross, 1998a), and that flexibility in emotion regulation choice may be particularly important (Bonanno et al., 2004; Sheppes et al., 2014).

Although tentative at this exploratory stage, abovementioned findings support the central premise of the study: written information can alter affective responses towards sound stimuli, which in turn are related to the inclination to use certain strategies to cope with said stimuli. Specifically, the results lend some evidence to the idea, previously presented by, for example, Sheppes et al. (2014), that greater negative valence would be related to greater use of disengaging strategies, such as mental suppression. Sheppes et al. have further argued that disengaging strategies can be beneficial in the short term, but prohibit deeper processing of the emotional stimulus. In contexts such as the one in our study, where an emotional stimulus will have a short-term disruptive effect, distraction or mental suppression may be adaptive strategies. However, mental suppression has previously been shown to have delayed and potentially adverse consequences in a number of settings (Wang et al., 2020), including sounds (Kolbeinsson et al., 2022). When regulating emotion in response to recurrently encountered negatively valued stimuli, these strategies may not have the desired effects, and other strategies (e.g., acceptance) are, therefore, likely to be more adaptive.

Regarding emotion regulation more generally, results suggest that participants used strategies that minimized effort while sufficiently regulating their affective response. Acceptance was the most commonly used of the four emotion regulation strategies, while cognitive reappraisal was the least common. Acceptance has previously been shown to be a resource-efficient strategy (Alberts et al., 2012), while reappraisal has been hypothesized to be a costly strategy (Sheppes et al., 2014). The results suggest that acceptance may be a preferred strategy when forced to perform a demanding task in the presence of an unavoidable, potentially distracting, sound stimulus. Additionally, it is important to note that most participants in the current study endorsed using multiple regulation strategies. As pointed out by others in recent years (e.g., Opitz et al., 2015; Szasz et al., 2018), emotion regulation is unlikely to involve only one strategy at a time, but is more likely a pattern of simultaneously employed strategies. The present results lend some additional support to this notion.

Concerning serial recall performance, no significant results emerged, suggesting that the sound did not distract performance on the task and that the manipulation did not influence auditory distraction. In contrast to more complex sounds, such as spoken words, noises such as the one used in the current study do not usually disrupt performance on serial recall (Tremblay et al., 2001). Negative emotional information might, however, have been expected to result in distraction, as previous studies have found a distractor-valence effect on the serial recall task when spoken distractor words are emotionally negative (Buchner et al., 2004; Marsh et al., 2018). While the lack of a valence effect in the current study may partly be a result of the manipulation only having a short-term impact, the results may also suggest that negative valence in itself may not be enough to cause distraction. Rather, induced emotional valence may enhance the ability of already disruptive stimuli to distract attention. As an example of this, Buchner et al. (2006) found increased distraction after having induced meaningless pseudowords with negative valence. However, Buchner et al. (2006) did not directly manipulate emotional valence, but rather associated certain pseudowords with a risk of loss, and there was no measure of emotional responding in the study. Using a methodology similar to ours, but adapted for measuring auditory distraction, could thus be beneficial in further isolating the specific effect of perceived emotional valence on auditory distraction, regardless of semantic meaning or sound characteristics.

Some limitations and procedural shortcomings should be mentioned. The present sample consisted of participants predominantly recruited from a student population. To assess generalizability, the results need to be replicated in a more representative sample. Potential applications could also be found in similar studies conducted with clinical samples, such as sufferers of tinnitus or hyperacusis*.* Similarly, only one type of sound was used in the current study. Artificial noises such as that used in the current study are rarely encountered in daily life and they are not inherently distracting. They are, however, uniquely suited for the purposes of the current study, as their acoustic properties allow them to be interpreted as either negative or positive, depending on contextual cues. Presently, we were mainly interested in isolating the component of verbal information, and including additional sound stimuli would potentially introduce an additional confounding factor. Replicating the current findings with inherently distracting sounds would reinforce the generalizability of the results and allow for the examination of potential interactions with the experimental manipulation.

Regarding the outcome measures, we did not collect any physiological or implicit measures of emotional responding. Subjective measures of emotional responding carry significant benefits in being easily recorded and providing valuable information on evaluative responding but are vulnerable to effects of social desirability. When provided with verbal information on how a sound is usually perceived, it is possible that participants would conform and thus rate the sound more in line with the information. The addition of response measures in a different domain (e.g., physiological measures), and a social desirability scale, could help elucidate the role of social desirability in studies of verbal information. In fact, there are previous examples, for example Field et al. (2001; Field and Lawson, 2003), where initial studies on verbal information used subjective measures as the primary outcome, whereas later studies included behavioral and implicit measures to gauge the robustness of the effect. Similarly, the current study provides initial evidence requiring corroboration from subsequent research using implicit measures of affective responses. Additionally, given that no previous study has examined the spontaneous use of emotion regulation strategies in the context of auditory stimuli, we modified a previously developed measure. Thus, as the measure has not been evaluated for this specific purpose, the results should be interpreted with some degree of caution.

Finally, the lack of a neutral control condition in the current study limits our ability to draw conclusions regarding the specific effects of the valenced instructions. For instance, we are not able to draw conclusions about how the manipulation impacted the use of regulatory strategies compared to participants' natural tendencies. The current study does, however, provide initial support for an effect of verbal information on stimulus evaluation and the use of emotion regulation strategies. Contrasting emotionally valenced instruction to a neutral control condition is an important next step in understanding the specific effects of such instructions.

In conclusion, the present study provides preliminary evidence that written information, in isolation, can influence participants' perceptions of a sound's emotional valence and that this, in turn, might influence how participants regulate auditory-induced emotion. While research has shown that instructions can alter emotional responses to a stimulus through verbal conditioning (e.g., Mertens et al., 2018) and that affective information can alter affective responding towards stimuli in non-auditory modalities (e.g., Field et al., 2001; Field and Lawson, 2003), we are aware of no previous study investigating the isolated effect of information on emotional responses and emotion regulation strategies in response to non-musical sound stimuli. While our preliminary findings are in need of replication, the study adds to the growing body of research on how sounds come to acquire emotional meaning and how individuals cope with emotional, task-irrelevant sounds by the use of emotion regulation strategies.

## Declarations

### Author contribution statement

Örn Kolbeinsson; Johan Wallqvist: Conceived and designed the experiments; Performed the experiments; Analyzed and interpreted the data; Wrote the paper.

Erkin Asutay: Conceived and designed the experiments; Analyzed and interpreted the data; Wrote the paper.

Hugo Hesser: Conceived and designed the experiments; Analyzed and interpreted the data; Wrote the paper.

### Funding statement

This work was supported by 10.13039/501100004359Vetenskapsrådet [DNR 2016–02283].

### Data availability statement

Data will be made available on request.

### Declaration of interests statement

The authors declare no conflict of interest.

### Additional information

No additional information is available for this paper.

## Appendix A Supplementary data

supplementaryHeliyon supplementaryHeliyon
