# Supplementary material for: Prior information can alter how sounds are perceived and emotionally regulated
Source: Heliyon. 2022 Jun 24;8(6):e09793. doi: 10.1016/j.heliyon.2022.e09793 (PMC9244733; doi:10.1016/j.heliyon.2022.e09793)
Supplement: supplementaryHeliyon [file mmc1.docx]

**Supplemental Materials**

When sounds threaten to distract: Prior information can alter how sounds are perceived and emotionally regulated

Submitted to: *Heliyon*

Contents

1. Adapted version of the SERI

**1. Adapted version of the SERI**

Think of the sound and which strategies you used to cope with it during the memory task. You will find a list of statements below. Circle the number which best represents how well you agree with each statement about how you coped with the sound. Please respond to each statement in the given order, without skipping anything.

Agree completely

Disagree completely

|  |  |  |  |  |  |  |  |  |
| --- | --- | --- | --- | --- | --- | --- | --- | --- |
| 1 | I tried to think of something other than the sound | 1 | 2 | 3 | 4 | 5 | 6 | 7 |
| 2 | I tried to think more positively of the sound | 1 | 2 | 3 | 4 | 5 | 6 | 7 |
| 3 | I tried to suppress the sound from my consciousness | 1 | 2 | 3 | 4 | 5 | 6 | 7 |
| 4 | When the sound appeared, I accepted it as it was | 1 | 2 | 3 | 4 | 5 | 6 | 7 |
| 5 | I tried to think of things unrelated to the sound | 1 | 2 | 3 | 4 | 5 | 6 | 7 |
| 6 | I tried to think of something positive about the sound | 1 | 2 | 3 | 4 | 5 | 6 | 7 |
| 7 | I tried to not think of the sound | 1 | 2 | 3 | 4 | 5 | 6 | 7 |
| 8 | I allowed the sound to come just as it was | 1 | 2 | 3 | 4 | 5 | 6 | 7 |
| 9 | I tried to think of something else instead of dealing with the sound | 1 | 2 | 3 | 4 | 5 | 6 | 7 |
| 10 | I tried to change my way of thinking about the sound | 1 | 2 | 3 | 4 | 5 | 6 | 7 |
| 11 | I tried to think the sound away | 1 | 2 | 3 | 4 | 5 | 6 | 7 |
| 12 | I let the sound be without being preoccupied with it or avoiding it | 1 | 2 | 3 | 4 | 5 | 6 | 7 |
| 13 | I tried to focus on the task instead | 1 | 2 | 3 | 4 | 5 | 6 | 7 |
| 14 | I tried to view the sound in a more positive way | 1 | 2 | 3 | 4 | 5 | 6 | 7 |
| 15 | I tried to pretend the sound was not there | 1 | 2 | 3 | 4 | 5 | 6 | 7 |
| 16 | I let the sound be without making efforts to change my experience of it | 1 | 2 | 3 | 4 | 5 | 6 | 7 |
